# Evaluation and modulation of bactericidal potential of different antibacterial agents against bacterial pathogens from conjunctivitis infections

**DOI:** 10.1186/s12348-025-00474-w

**Published:** 2026-03-06

**Authors:** Saman Arshad, Nazish Mazhar Ali, Sidra Munir, Mariam Wasif, Maham Chaudhry, Bushra Mazhar, Muhammad Ahsan Raza

**Affiliations:** 1 Department of Zoology, Government College University, Lahore, Pakistan; 2 Institute of Zoology, Punjab University, Lahore, Pakistan

**Keywords:** Conjunctivitis, Plant extracts, Nanoparticles, Antibiotics, Resistance, Biochemical characterization

## Abstract

Conjunctivitis is one of the most common eye infections seen in hospitals. Youngsters under the age of seven, women at age 22 and men at age 28 are most likely to be diagnosed with this infection. The current study was designed to identify and characterize the conjunctivitis associated pathogens and to evaluate their sensitivity or resistance against commonly used antibiotics (metronidazole, ciprofloxacin, azithromycin, and levofloxacin), aqueous plant extracts (*Ficus religiosa, Syzygium cumini, Azadirachta indica, Allium cepa, Eucalyptus camaldulensis, Syzygium aromaticum, Aloe barbadensis*, and *Citrus limon*) and green synthesized silver nanoparticles. The samples were taken at Fatima Memorial Hospital, Lahore. Blood agar test was used for screening of alpha and beta hemolytic bacterial pathogens. Antibacterial activity against pathogenic isolates was done using well diffusion method. Antibiotics showed range of bactericidal potential against pathogens considerably more effective against isolated bacteria. Maximum antibacterial activity against bacterial strains was observed in *E. camaldulensis*, *S. cumini*, and *C. limon* (13.66 ± 1.20 to 9 ± 0.57, 15.5 ± 0.76 to 10.33 ± 1.45, and 21.33 ± 0.88 to 12.66 ± 0.33 respectively). Green synthesized silver nanoparticles showed better results as antibacterial agents with the zones of inhibition measuring 11.66 ± 0.66 to 8.83 ± 0.72; 12.5 ± 0.62 to 9.83 ± 0.72; 16.16 ± 1.09 to 10.83 ± 1.01; 13.33 ± 1.20 to 8.83 ± 0.72; 12.16 ± 1.16 to 7.33 ± 0.66, and 13.16 ± 0.59 to 8.33 ± 0.88 respectively. Biochemical and molecular characterization of pathogens was done. Bacterial strains were identified as *Bacillus thuringiensis*, *Bacillus cereus*, *Bacillus paramycoides*, *Bacillus coahuilensis* and *Pseudomonas aeruginosa*. This study showed that because of mis- use or over use of antibiotics, pathogens have developed resistance. So conventional medication may be replaced by biological antibacterial tools such as plant extracts and green synthesized silver nanoparticle.

## Introduction

Conceivably the most delicate organ in the human body is the eye [12]. Conjunctivitis is a common condition found in ophthalmology clinics worldwide [14]. Conjunctivitis is typified by inflammation of the conjunctival tissue, ocular discharge, and vascular enlargement. It could be infectious or not, acute or chronic, etc. Allergies, viruses, and bacteria can all cause conjunctivitis [27]. The months with the highest frequency of bacterial conjunctivitis are December through April [4, 47]. Common bacterial infections are usually the cause of bacterial conjunctivitis. These pathogens include *Streptococcus pneumoniae, Staphylococcus* spp*., Haemophilus* spp.*, Moraxella* spp*.* [10]*,* and. Most cases of bacterial conjunctivitis resolve on their own in 1 to 2 weeks. Children are impacted by bacterial conjunctivitis far more frequently than adults [11]. Youngsters under the age of seven are most likely to be diagnosed, and the age range between 0 and 4 years old is of greatest importance. The second highest point in the distribution is reached by women at age 22 and men at age 28.

Since antibiotic resistance causes millions of deaths worldwide, it poses a serious threat to public health. Antibiotic resistance has become frighteningly widespread in recent years [28]. Antibiotic resistance is a major global health concern, since it is estimated to have caused at least 1.27 million deaths worldwide and roughly 5 million deaths in 2019 [19]. The urgent need for safer and more effective agents stems from the growing burden of microbial resistance and the adverse effects of synthetic medications on global health and death rates. The pursuit of this project has intensified the hunt for alternatives to plant extracts and their nanoparticles [20].

According to their diameters, nanoparticles are the end product of technologically modifying matter and are a few orders of magnitude larger than an atom as a result of molecular processing of matter [56]. Au, Ag, Cu, Ni, Si, and Se are examples of metal NPs. AgNPs are a well-researched nanomaterial that can be produced chemically or biologically, primarily using plants and microbes [32]. The medical field has shown that AgNPs' antibacterial and anti-inflammatory properties are helpful in managing microbial infections [16, 38]. Using the conventional disk diffusion experiment, Yassin et al. [54, 55] demonstrated the synergistic antibacterial efficacy of the biosynthesized AgNPs with the antibiotic colistin against multidrug resistant bacterial including *Acinetobacter baumannii, Enterobacter cloacae*, *E. coli*, *Klebsiella pneumoniae*, *Salmonella typhimurium* and *P. aeruginosa*. Yassin et al. [54, 55] performed disc diffusion method by using green synthesized AgNPs of *Origanum majorana* aqueous leaf extract to check their antibacterial efficiency against multidrug resistant bacterial strains. In this study GC–MS was carried out to see the most effective constituents having antibacterial activity. Similarly, Aljeldah et al. [6] detected synergistic efficiency of AgNPs and the antibiotic fosfomycin against, *Klebsiella pneumoniae*, Methicillin-resistant *Staphylococcus aureus*, *Escherichia coli* and *Enterobacter cloacae* strains. Maniah et al. [35] studied the effects of green synthesize AgNPs from the seeds of *Trigonella foenum-graecum* (fenugreek).

AgNPs' effects on microorganisms are explained by a number of theories, including as the inhibition of enzymes required for cell life, the activation of enzymes required for cell survival, the increase in cell permeability, and the penetration of silver ions into cells. AgNPs interact with the phosphate and sulfur of bacterial DNA to form a byproduct of microbial death. According to Wang et al. [30], nanoparticles stop signal transduction and stop bacteria from growing. The utilization of diverse bio-based materials sourced from bacteria, fungi, plants, and algae for the manufacturing of nanoparticles has stimulated the development of pragmatic, eco-friendly, economical, and easily expandable techniques [17].

The WHO recommends incorporating herbal medicines into national health care programs since they are safer, more widely available, and less expensive than modern synthetic treatments [24]. In affluent countries, conventional medicine derived from medicinal plants is used by about 80% of the population [29, 50]. Certain antibacterial substances can be found in plants, spices, and herbs. Aloe Vera leaves have an inside gel that may be rich in bioactive materials such as vitamins, minerals, enzymes, amino acids, and polysaccharides. Aloe vera is a multipurpose plant with numerous medical applications because of its anti-inflammatory, antioxidant, antibacterial, and antiviral qualities [18]. Citric acid [34], secondary metabolites [25], phenolic derivatives [42], and a host of other chemicals that exhibit broad antibacterial activity comprise the majority of citrus fruits [50].

Flavonoids are found in citrus fruits like lemons in addition to alkaloids [42]. *Syzygium aromaticum*, the clove tree, is native to Indonesia. Cloves are mostly composed of the phenolic chemicals flavonoids, hydroxoxibenzoic acid, hydroxicinamic acid, and hydroxoxiphenyl propense. Additionally, higher gallic acid concentrations were found [45]. Due to their high phytonutrient content, which includes flavonoids and anthocyanins [7], onions have antibacterial, anti-inflammatory, antifungal, anticancer, and antioxidant qualities [3]. The majority of jamun leaves' health benefits are attributed to phytochemicals such gallic acid, flavonoids, tannins, mallic acid, jambolin, essential oils, jambosine, ellagic acid, betulinic acid and antimellin that are present in jamun leaves [33]. The leaves of *F. religiosa* contain tannins, terpenoids, flavonoids, and other phytochemicals [48]. It is well recognized that Eucalyptus members serve as significant repositories of a diverse array of secondary metabolites, numerous of which possess several biological functions [57].

The current research work was aimed to isolate and screen the conjunctivitis associated bacterial pathogens. To evaluate the antibacterial activity of a variety of antibiotics, plant extracts and green synthesized silver nanoparticles (AgNPs). Also to perform biochemical and molecular characterization of isolated pathogenic bacterial strains from conjunctivitis.

## Materials and methods

### Sampling

Five samples of conjunctivitis eye infection were collected from Fatima Memorial Hospital (FMH), Shadman Lahore by using sterilized culture sticks. Further study was done in microbiology research laboratory of Zoology department in Government College University, Lahore.

### Bacterial isolation

**Fig. 1:**
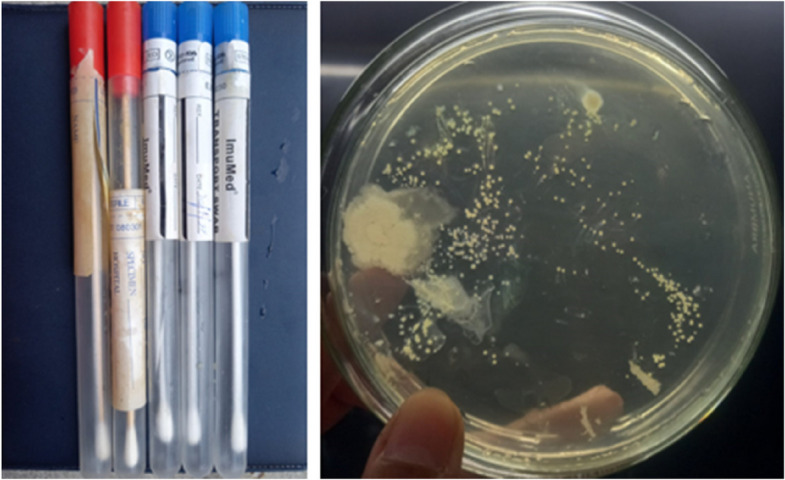
Bacterial sampling sticks (left), Spreading of samples (right)

**Fig. 2:**
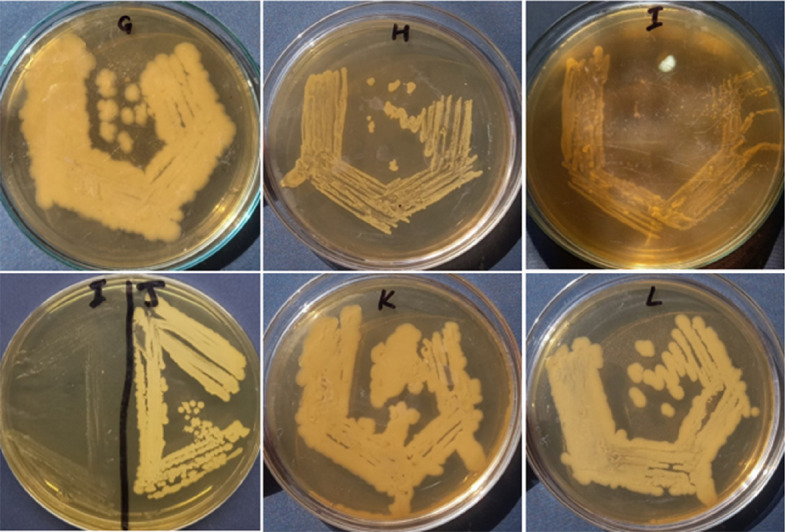
Quadrant method streaking of six isolated bacterial strains G, H, I, J, K, L

For isolation of bacteria from culture sticks, 2.8% Nutrient agar medium (2.8 g nutrient agar/100 ml distilled water) was prepared and autoclaved at 121 ºC temperature and 15 psi pressure for 60 min. Autoclaved nutrient agar medium was poured into sterile petri plates inside sterilized laminar air flow. Spread plate method was used to isolate bacteria. After solidification of nutrient agar in petri plates, collected samples were spread on nutrient agar plates with the help of culture sticks and sterilized glass spreader and plates were incubated for 24 h at 37 ºC. After isolation, bacterial isolates were purified by using streak plate method (Figs. 1 and 2).

### Pathogenicity test and biochemical characterization

**Fig. 3:**
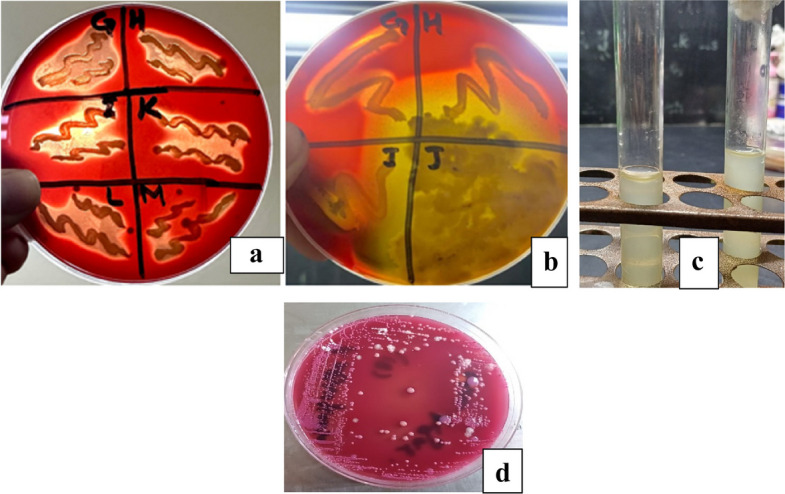
**a** & **b** Pathogenicity test, **c** Pyoverdin production test (yellow greenish ring), **d** MacConkey agar test

Blood agar test was performed to check the pathogenicity of isolated bacterial strains. 6 ml human blood was added in 2.8% autoclaved nutrient agar. Bacterial strains were streaked on already prepared blood nutrient agar plates and incubated at 37 ºC for 24 h. After incubation, blood hemolysis around bacterial streaks were observed. Greenish zone indicated α-hemolysis, clear zone indicated β-hemolysis and no zone indicated γ-hemolysis (non-pathogenic). After pathogenicity test, glycerol stocks of pathogenic bacteria were prepared for future use Different biochemical tests such as utilization of MacConkey agar media, cetrimide agar media, pyoverdin production test was also done after the confirmation of *P.aeruginosa* on cetrimide agar plates was done. Other tests such as catalase, citrate, and motility tests were also done. Gram staining was also done to check the shapes of bacterial pathogens (Fig. 3) [51].

### Antibacterial activity of antibiotics, aqueous plant extracts and green synthesized silver nanoparticles (AgNPs)

**Fig. 4:**
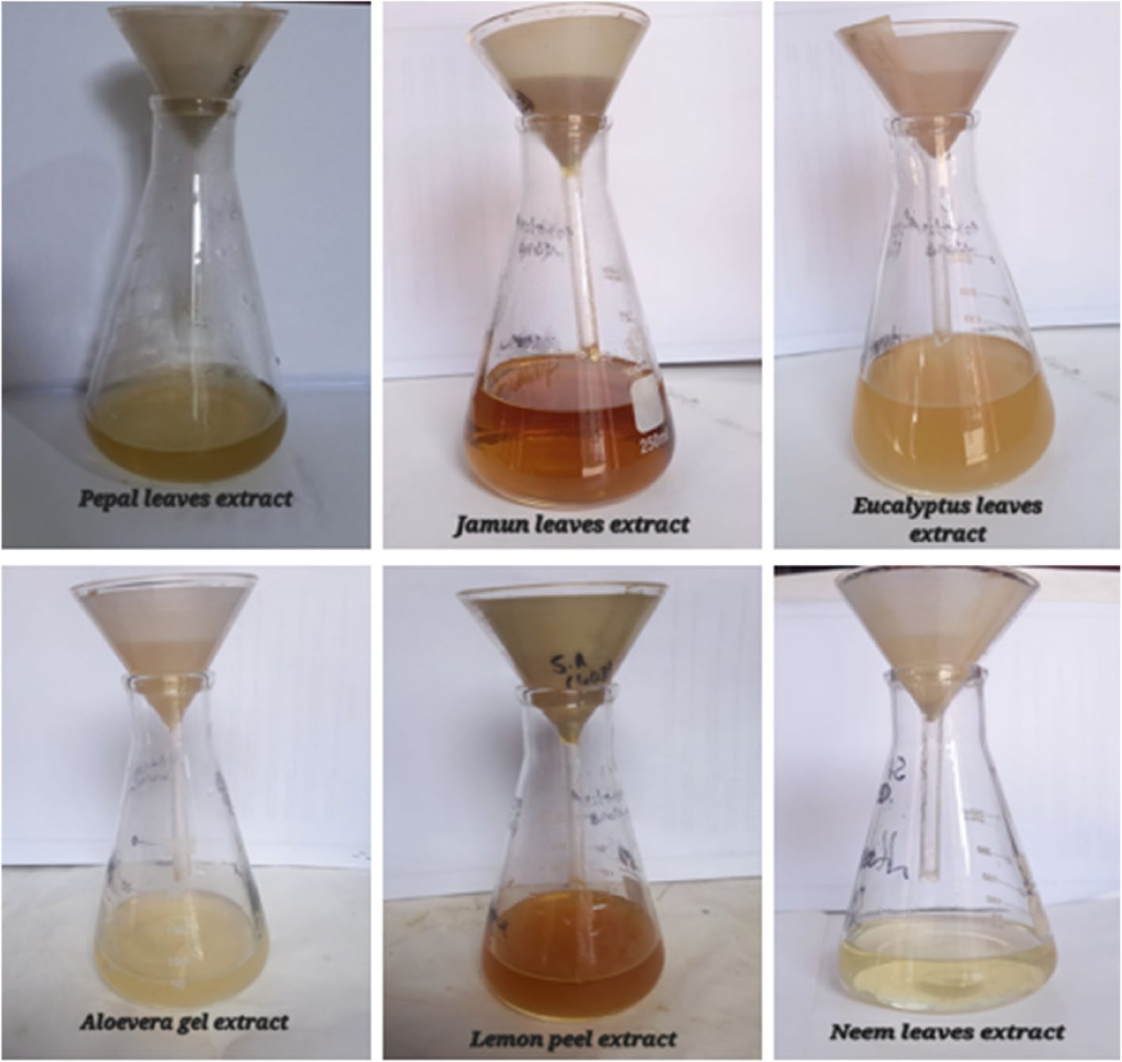
Aqueous plant extracts

**Fig. 5:**
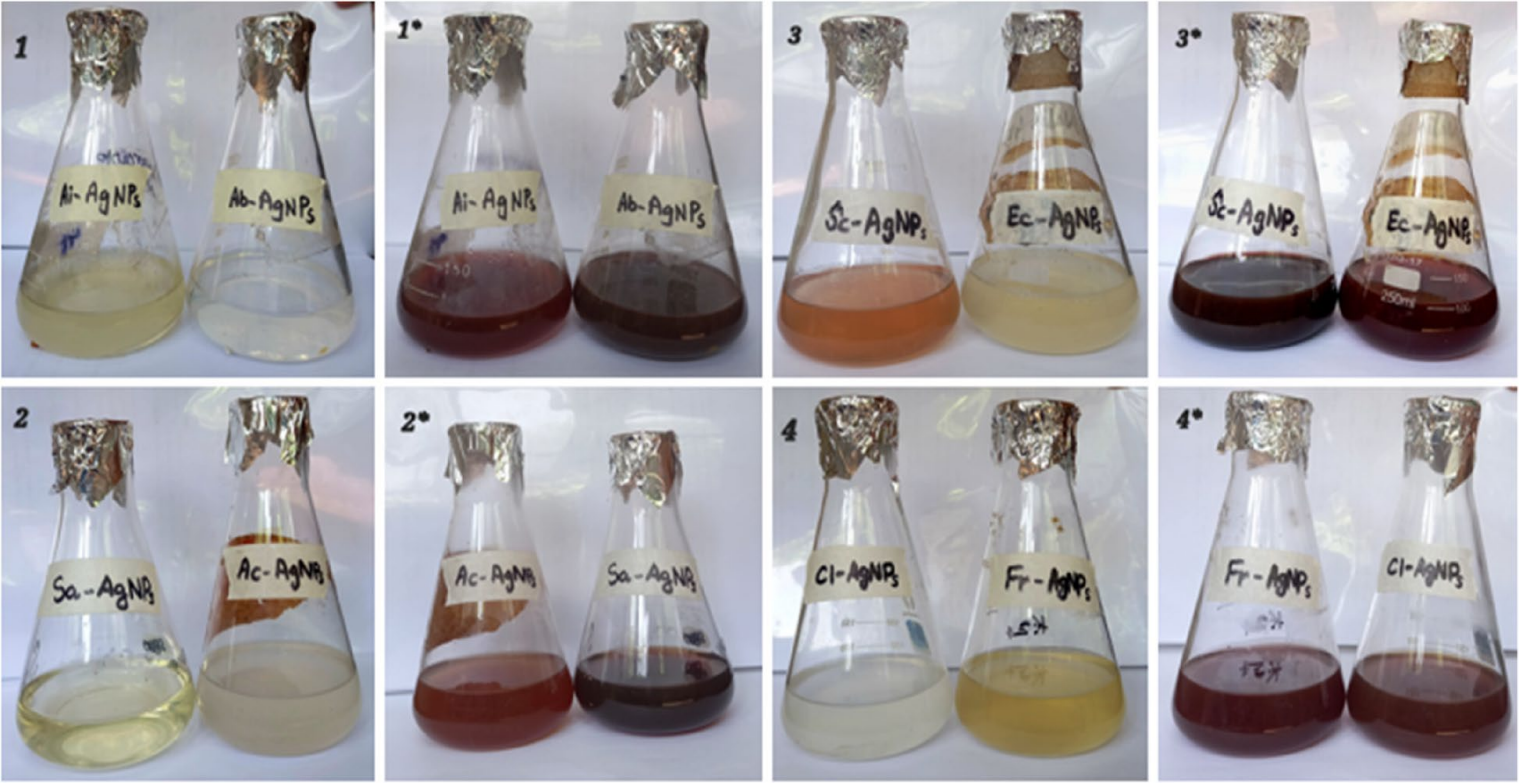
Synthesis of silver nanoparticles, (1, 2, 3, 4 before sunlight exposure and 1*, 2*, 3*, 4* after sunlight exposure)

**Fig. 6:**
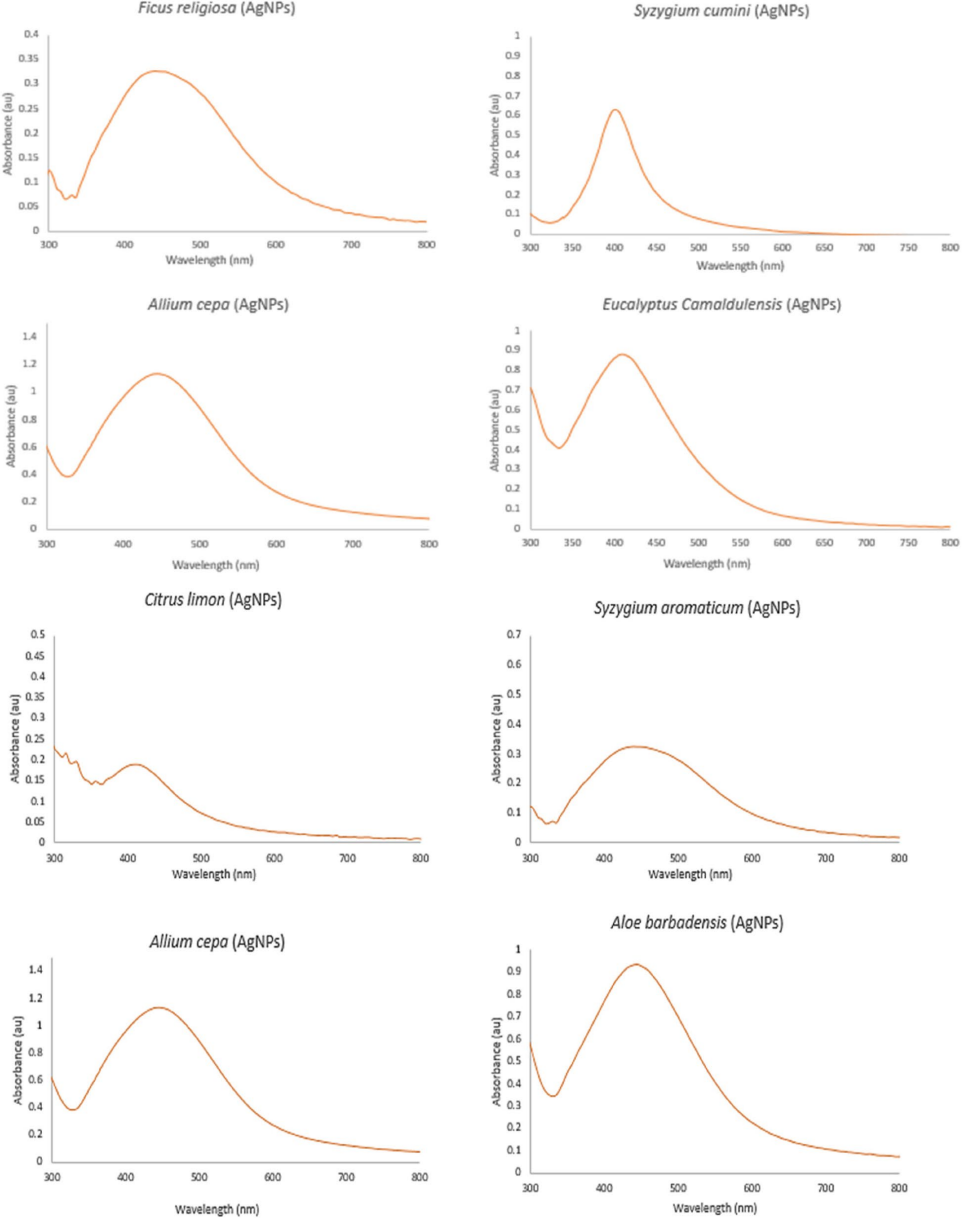
UV visible spectrophotometry of green synthesized AgNPs

**Fig. 7:**
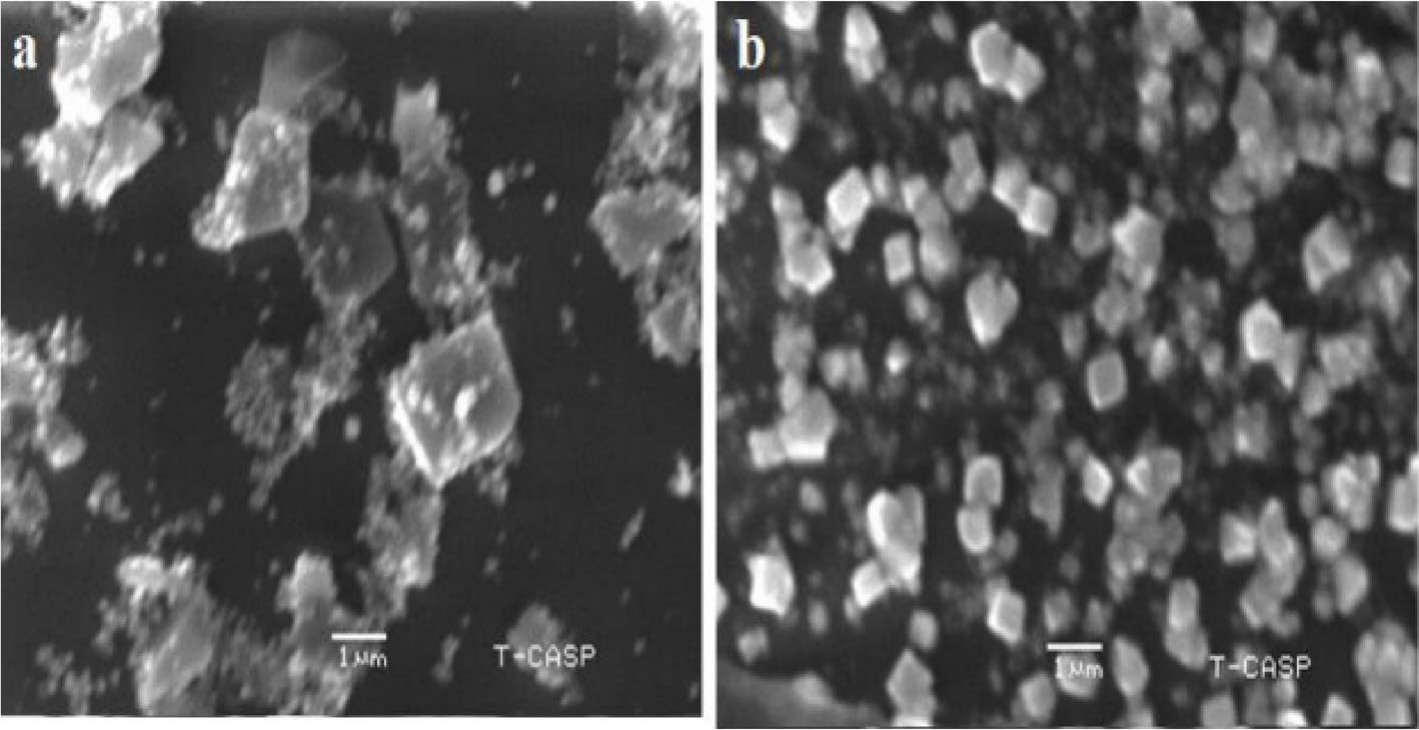
SEM of silver nanoparticles at different magnifications (17nm size)

**Fig. 8:**
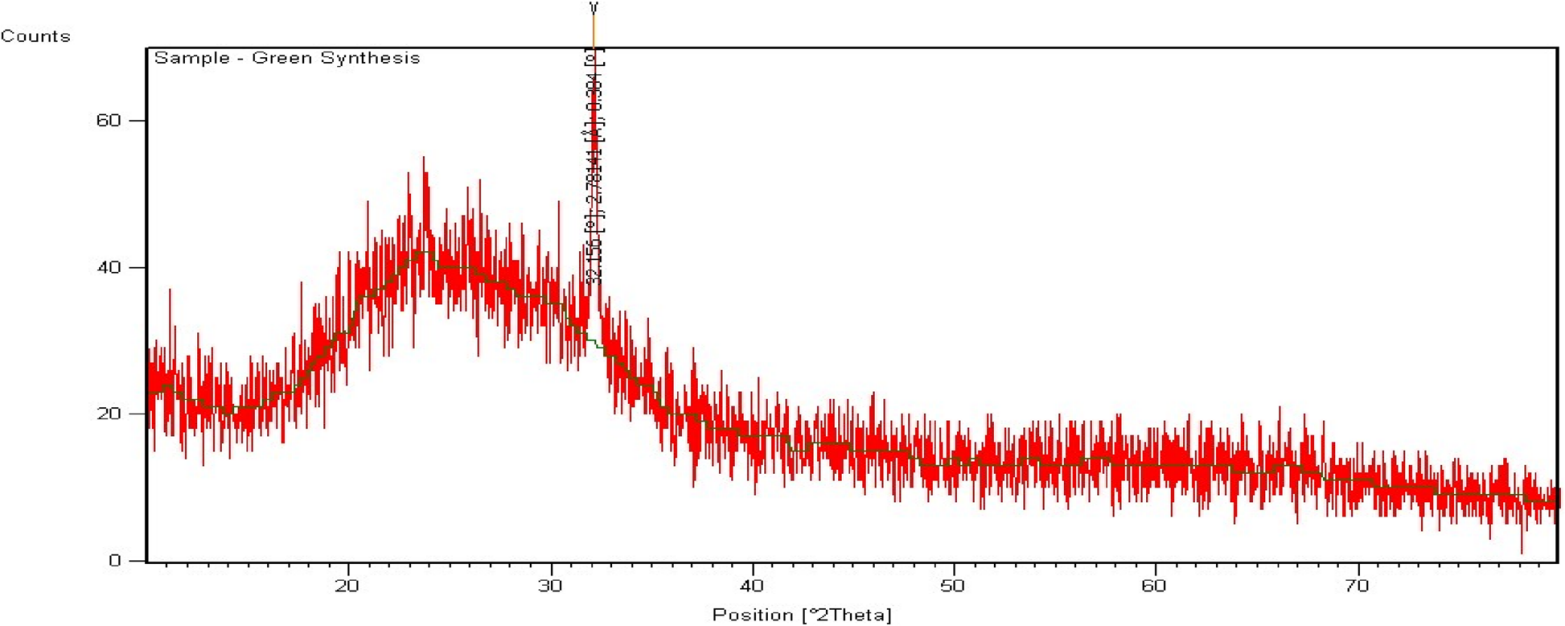
XRD chromatogram of silver nanoparticles green synthesized

To check antibacterial activity of antibiotics, aqueous plant extracts and green synthesized silver nanoparticles, and resistance of bacteria against them, well diffusion method was used. For antimicrobial sensitivity test (AST) guidelines of Clinical and Laboratory Standards Institute (CLSI) were followed [53]. For this purpose, four antibiotic (metronidazole, levofloxacin, azithromycin and ciprofloxacin) solutions were prepared by solubilizing 10 mg antibiotic in 50 ml distilled water. Eight different types of aqueous plant extracts were synthesized by heating 40 g of plant medium (leaves/seeds/peel/gel/bulb) in 200 ml distilled water, at 50 ⁰C for 20 min. These extracts included *A. indica, S, cumini, E. camaldulensis, F. religiosa* (leaf extract), *C. limon* (peel extract), *A. barbadensis* (gel extract), *S. aromaticum* (seed extract) and *A. cepa* (bulb extract). After heating, extracts were filtered (whattman filter paper) and stored in refrigerator (Figs. 4 and 5). For preparation of green synthesized AgNPs, biological method was used. 100 ml silver nitrate solution (0.017 g AgNO_3_/100 ml distilled water) was prepared. After that, 10 ml of already prepared aqueous plant extract was mixed with 90 ml of AgNO_3_ solution, covered the opening of flask and placed in sunlight for 10 min to observe colour change. Colour change from extract colour to dark reddish brown was the indication of AgNPs formation. All nanoparticles were covered with aluminum foil and stored in refrigerator. For the confirmation of nanoparticle formation, UV–visible spectrophotometry was performed. Peak should be in the range of 350–600 nm (Fig. 6). Scanning electron Microscopy (SEM) (Fig. 7) and X-ray Crystallography (XRD) (Fig. 8) for nanoparticles was also done.

For well diffusion method, nutrient agar plates were prepared by pouring autoclaved nutrient agar. 5 wells were formed on a single plate and bacteria were spread on the agar plate. 50 μl of antibiotic solutions, aqueous plant extracts and AgNPs were poured in the well. Distilled water was used as a negative control. After incubation at 37 ⁰C for 24 h, zones of inhibition were observed and measured. Clear zones indicated susceptibility of bacteria while no zones indicated resistance of bacteria (Fig. 4).

### Molecular characterization of pathogenic strains

Phenol chloroform DNA extraction method was utilized for extraction of bacterial DNA for ribotyping. Genes were amplified by commonly used PCR technique and products were sent for purification and sequencing [21].

### Statistical analysis

To compare the control group's values with those of the treatment groups (antibiotics, plant extracts, and green synthesized nanoparticles), one-way ANOVA was performed using Graph Padprism (Version 9.0) software, followed by Tukey's post-hoc test. Every value was displayed as a graph with the mean and standard error mean (SEM).

## Results

In the present study, different bacteria were isolated from samples of conjunctivitis infection, cultured in microbiology laboratory of Government College University, Lahore and observed their resistance against different antibiotics, aqueous plant extracts and green synthesized silver nanoparticles. These bacterial strains were identified by biochemical characterization as *Bacillus thuringiensis* (strain-H), *Bacillus paramycoides* (strain-I), *Pseudomonas aeruginosa* (strain-J), *Bacillus coahuilensis* (strain-K), and *Bacillus cereus* (strain-L).

*Bacillus* is a rod-shaped, gram-positive, motile bacterium that is widely found in nature and forms spores [12]. *B. thuringiensis* and *Bacillus cereus* are food pathogens and can cause vomiting and diarrhea [41], systemic infections like meningitis and bacteremia, as well as localized infections such infections of the ear canal and eyes [15]. *P. aeruginosa* is a gram negative, lactose-fermenting and non-fermenting bacteria [13, 36]. It is a common rod-shaped [43] opportunistic pathogen possessing a broad range of adaptable virulence factors. The 16 s rRNA has also been used to identify *Pseudomonas* sp. as a component of the conjunctival microbiome. Acute conjunctivitis [44], dacryocystitis [31], post-surgical and post-traumatic endophthalmitis are all brought on by *P. aeruginosa* [12].

**Fig. 9:**
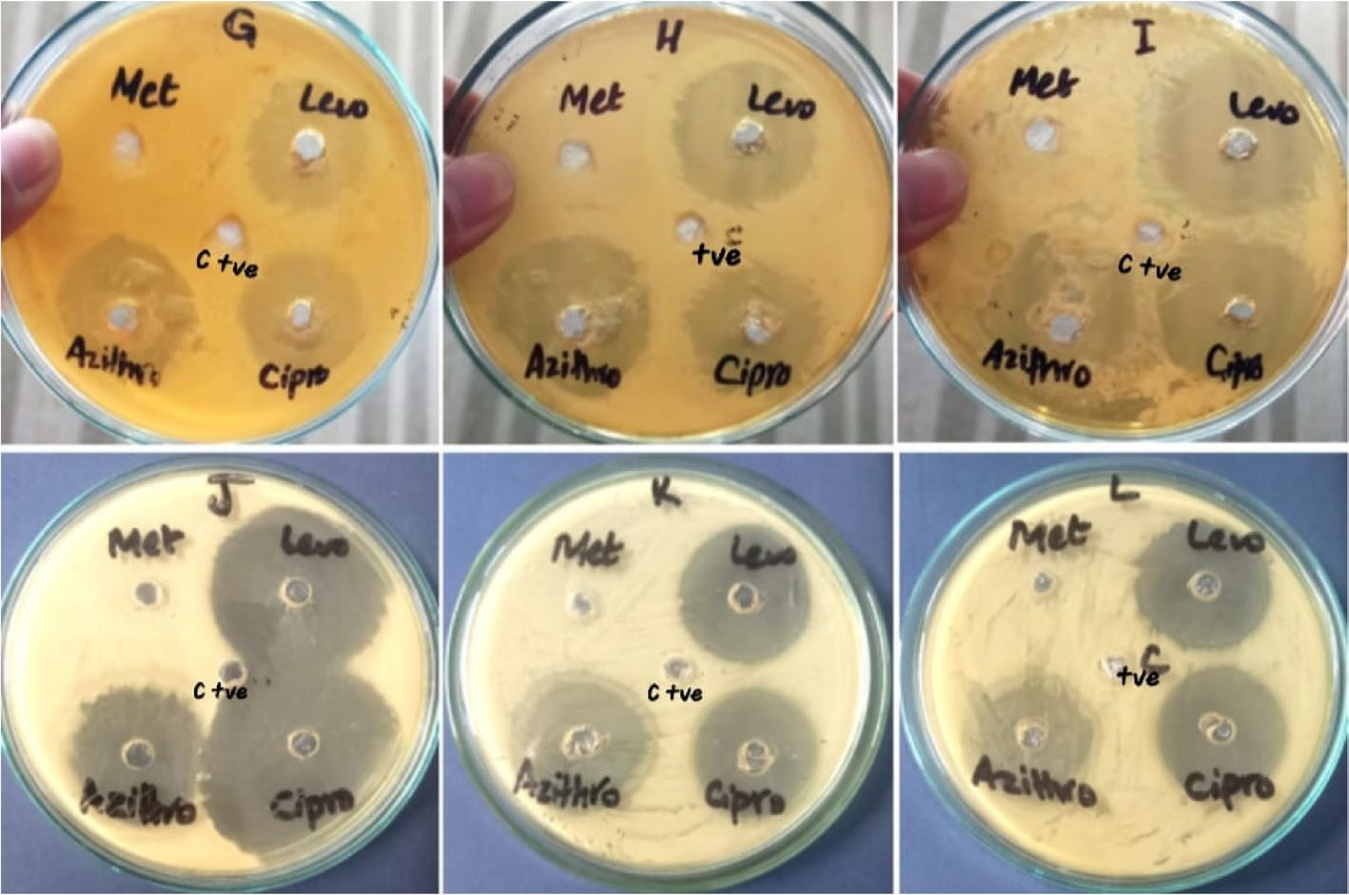
Antibacterial activity of antibiotics by well diffusion method (C+ve = positivecontrol)

**Fig. 10:**
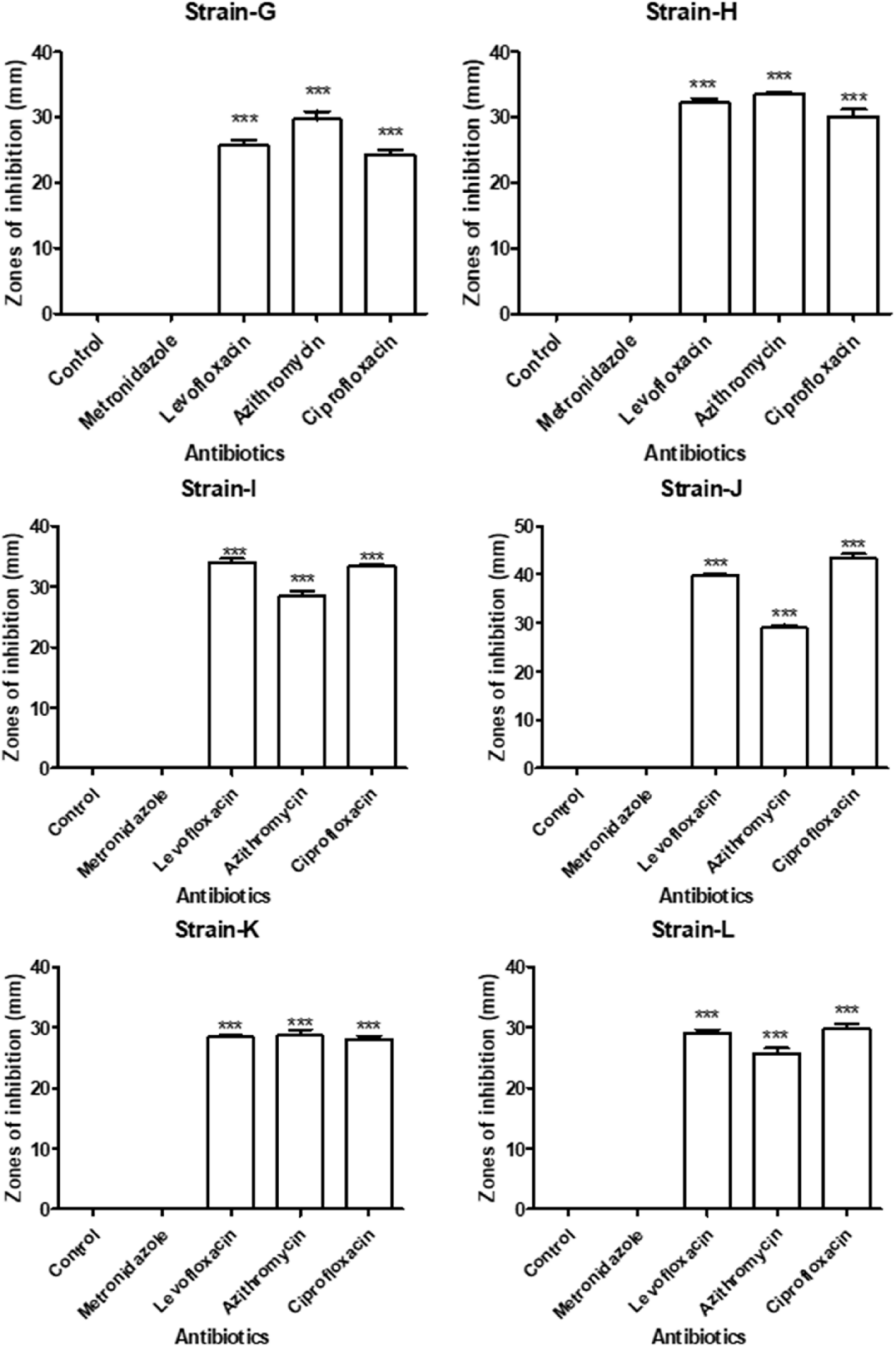
Antibacterial activity of antibiotics against bacterial strains, *** shows significant difference of antibacterial activity between control and other groups

Table 1 indicates antibacterial activity of 4 different kinds of antibiotics i.e. azithromycin, levofloxacin, ciprofloxacin and metronidazole with 50 μl concentration against isolated bacterial strains. All bacteria show resistance against metronidazole. All bacterial strains are sensitive to levofloxacin, azithromycin and ciprofloxacin. All the strains show least sensitivity against levofloxacin except *P. aeruginosa* with 39 mm zone of inhibition (ZOI). *B. thuringiensis* is highly susceptible to azithromycin with 33 mm ZOI followed by *P. aeruginosa* with 29 mm ZOI then *B. coahuilensis* and *B. paramycoides* with 28 mm ZOI and *B. cereus* is least sensitive with 25 mm ZOI. In case of ciprofloxacin, *P. aeruginosa* is highly sensitive with 43 mm ZOI while *B. coahuilensis* is least sensitive with 28 mm ZOI (Figs. 9 and 10).

**Table 1:** Antibacterial activity of antibiotics

Antibiotics	Zone of inhibition (mm) ± S.E				
	*Bacillus thuringiensis*	*Bacillus paramycoides*	*Pseudomonas aeruginosa*	*Bacillus coahuilensis*	*Bacillus cereus*
Metronidazole	R	R	R	R	R
Levofloxacin	32 ± 0.72	34 ± 0.57	39.66 ± 0.5	28.33 ± 0.33	29 ± 0.57
Azithromycin	33 ± 0.28	28 ± 0.88	29 ± 0.57	28.66 ± 0.88	25.66 ± 0.88
Ciprofloxacin	30 ± 1.01	33 ± 0.33	43.33 ± 0.88	28 ± 0.57	29.66 ± 0.88

*R* Resistant

Table 2 shows antibacterial activity of aqueous plant extracts against isolated bacterial strains. All bacterial strains are resistant to aqueous extracts of *A. indica*, *F. religiosa* and *A. barbadensis*. *P. aeruginosa*, *B. coahuilensis* and *B. cereus* show resistance against aqueous extracts of *A. cepa* and *S. aromaticum*. Out of *S. cumini*, *E. camaldulensis* and *C. limon* aqueous extracts, *C. limon* shows maximum antibacterial activity against *B. thuringiensis* (21 mm), *B. coahuilensis* (14.33 mm) and *B. paramycoides* (14 mm). While *S. cumini* aqueous extract is most effective against *P. aeruginosa* (13 mm), *B. coahuilensis* (15 mm) and *B. cereus* (13 mm).

**Table 2:** Antibacterial activity of aqueous plant extracts

Aqueous plant extracts	Zones of inhibition (mm) ± S.E				
	*Bacillus thuringiensis*	*Bacillus paramycoides*	*Pseudomonas aeruginosa*	*Bacillus coahuilensis*	*Bacillus cereus*
*Azadirachta indica*	R	R	R	R	R
*Syzygium cumini*	14.66 ± 0.88	12.16 ± 0.60	12.83 ± 0.6	15.5 ± 0.76	13.16 ± 0.60
*Eucalyptus camaldulensis*	13.66 ± 1.20	11 ± 1.15	9.83 ± 0.44	10.66 ± 0.88	11.16 ± 0.44
*Ficus religiosa*	R	R	R	R	R
*Aloe barbadensis*	R	R	R	R	R
*Allium cepa*	9.33 ± 0.33	11.66 ± 0.66	R	R	R
*Syzygium aromaticum*	10 ± 0.57	R	R	R	R
*Citrus limon*	21.33 ± 0.88	14 ± 0.57	12.66 ± 0.33	14.33 ± 0.33	12.66 ± 0.88

*R* Resistant

**Fig. 11:**
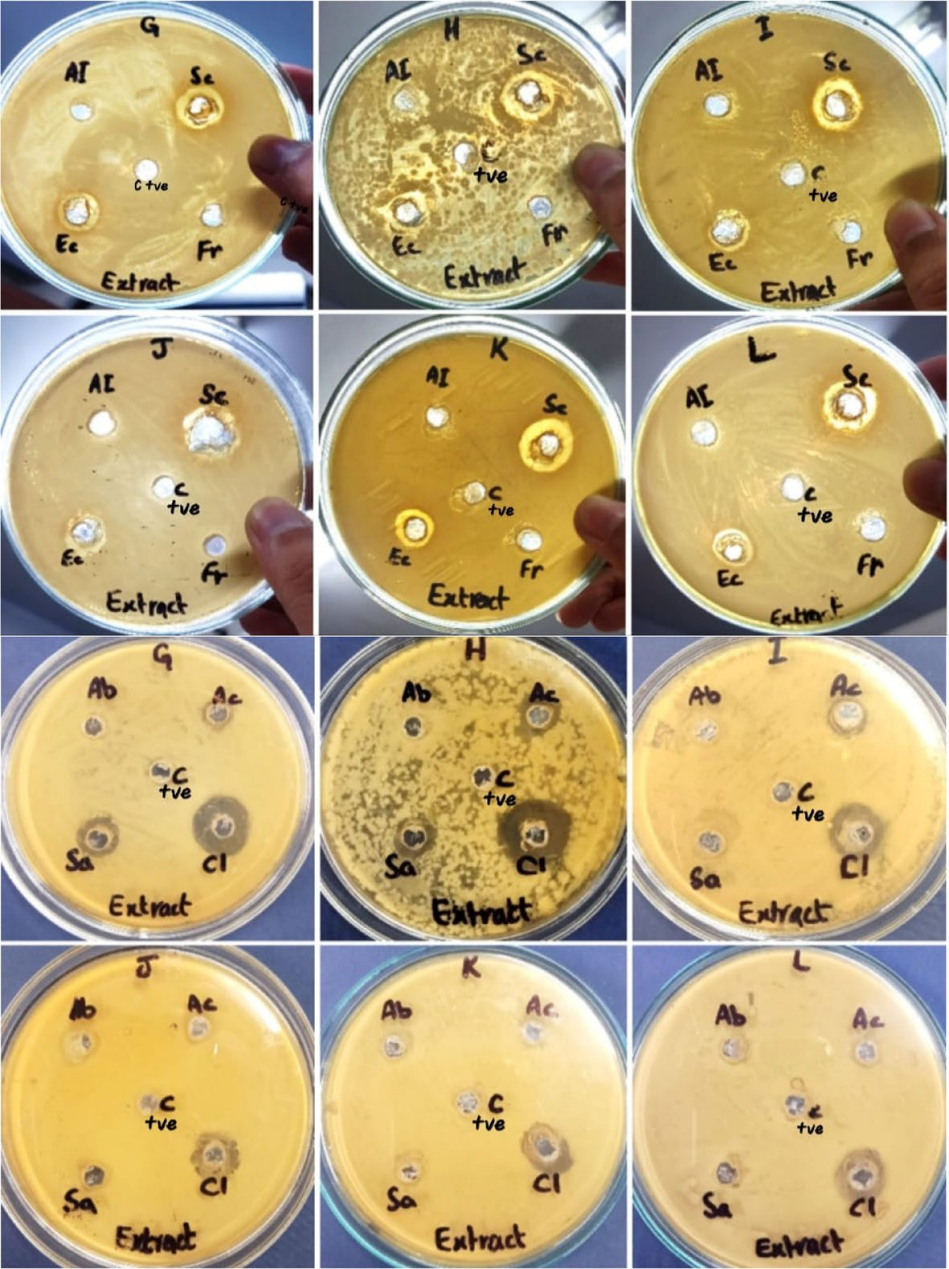
Antibacterial activity of aqueous plant extracts by well diffusion method (C+ve = positivecontrol)

**Fig. 12:**
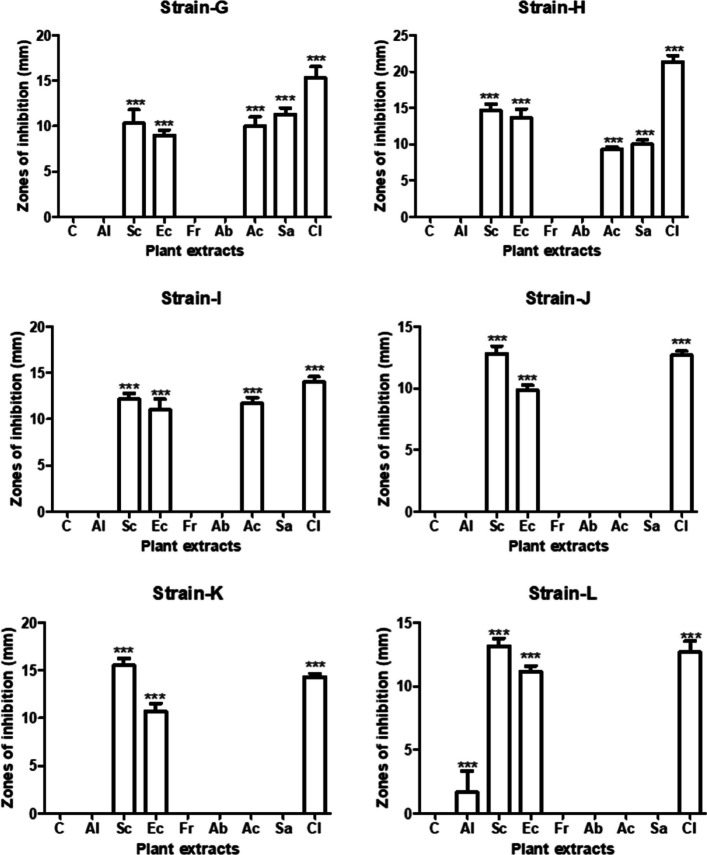
Antibacterial activity of aqueous plant extracts against 6 bacterial strains, *** shows significant difference of antibacterial activity between control and other groups

Table 3 indicates antibacterial activity of green synthesized silver nanoparticles (AgNPs of 17 nm size) against all isolated bacterial strains According to results, *A. indica*, *F. religiosa, S. aromaticum* and *C. limon* green synthesized AgNPs are effective against all isolated bacterial strains. *C. limon* AgNPs show maximum antibacterial activity against all bacterial strains. *B. thuringiensis* (16 mm) is highly sensitive to *C. limon* AgNPs followed by *B. coahuilensis* (14 mm), *B. paramycoides* (13 mm), *P. aeruginosa* (12 mm), and *B. cereus* (11 mm). While *E. camaldulensis* AgNPs gave ZOI of 7 mm against *B. cereus*. Out of all bacterial strains, *P. aeruginosa* is resistant to *A. barbadensis* and *A. cepa* AgNPs. All bacterial strains are resistant to only *S. cumini* AgNPs (Figs. 11 and 12).

**Table 3:** Antibacterial activity of green synthesized silver nanoparticles (Figs. 13 and 14)

AgNPs	Zones of inhibition (mm) ± S.E				
	*Bacillus thuringiensis*	*Bacillus paramycoides*	*Pseudomonas aeruginosa*	*Bacillus coahuilensis*	*Bacillus cereus*
*Azadirachta indica*	13.33 ± 1.20	11.66 ± 0.27	10 ± 1.01	10.66 ± 1.20	8.83 ± 0.72
*Syzygium cumini*	R	R	R	R	R
*Eucalyptus camaldulensis*	R	R	R	R	7.5 ± 0.28
*Ficus religiosa*	12.16 ± 1.16	12 ± 0.47	13 ± 1.15	12.33 ± 1.45	7.33 ± 0.66
*Aloe barbadensis*	11.66 ± 0.66	9.66 ± 0.72	R	8.83 ± 0.72	9.83 ± 0.60
*Allium cepa*	13.33 ± 0.88	13.16 ± 0.59	R	9.33 ± 0.33	8.33 ± 0.88
*Syzygium aromaticum*	10.83 ± 0.92	12.5 ± 0.62	12 ± 1.01	11.66 ± 1.20	11.66 ± 0.88
*Citrus limon*	16.16 ± 1.09	13 ± 0.47	12 ± 0.57	14 ± 1.15	10.83 ± 1.01

**Fig. 13:**
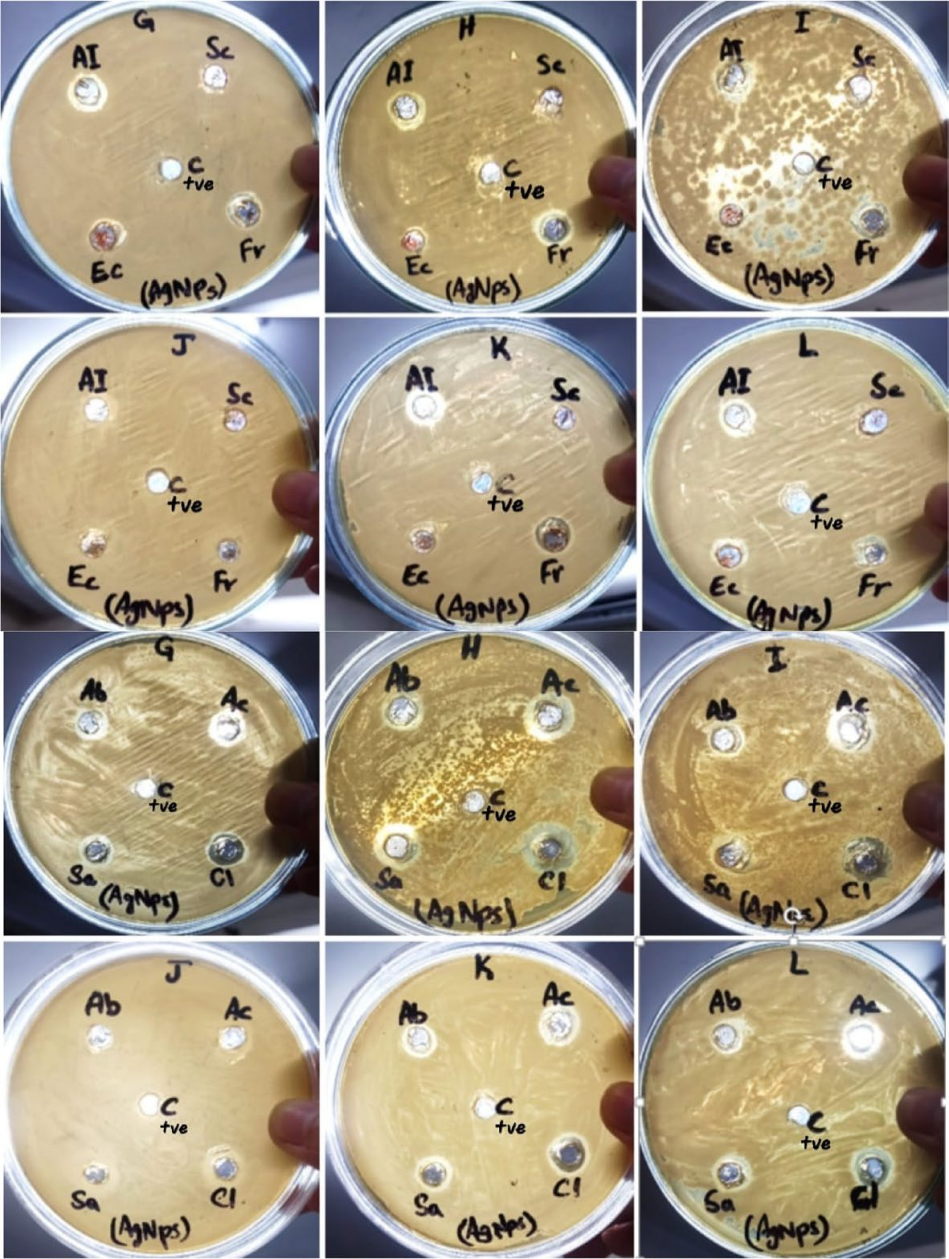
Antibacterial activity of green synthesized AgNPs by well diffusion method (C+ve = positivecontrol)

**Fig. 14:**
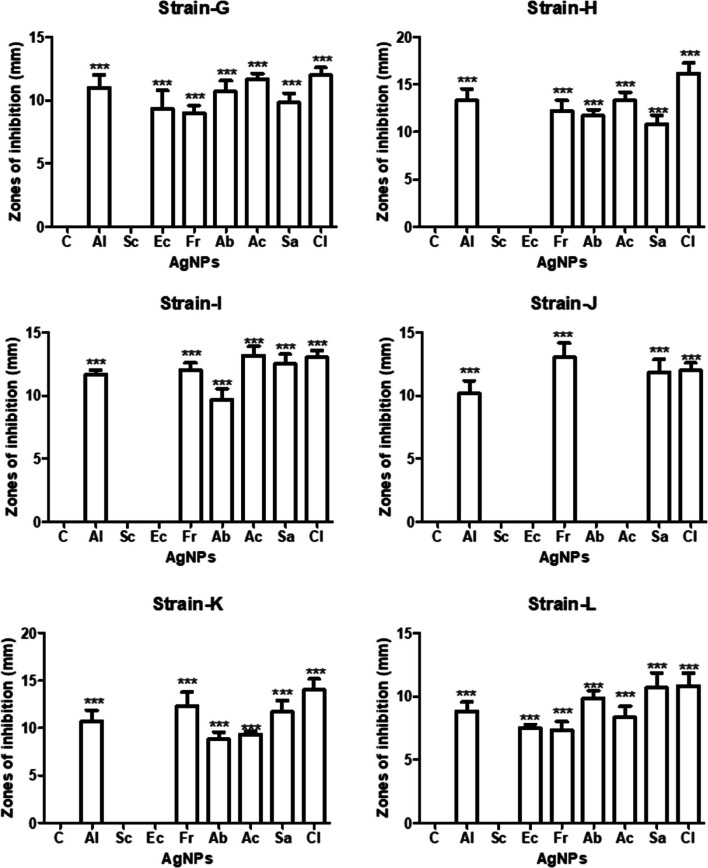
Antibacterial activity of green synthesized AgNPs against 6 isolated bacterial strains, *** shows significant difference of antibacterial activity between control and other groups

### Statistical analysis

By performing one-way ANOVA and Tukey’s post-hoc test, a clear notable difference between antibacterial activity of control and other groups was observed as shown in graphs (Figs. 10, 11, and 12). These graphs show antibacterial activity of different groups compared with control group.

### Molecular characterization

The FASTA sequence of 16S rRNA gene of all isolated strains were obtained after sequencing from Macrogen South Korea. Blast tool of NCBI was used to compare the sequence with previously reported sequences of 16S rRNA (Table 4).

**Table 4:** Accession number of pathogenic strains

Serial No	Strain	Species identified	Accession number
1	Strain H	*Bacillus thuringiensis*	PQ762144
2	Strain I	*Bacillus paramycoides*	PQ761549
3	Strain J	*Pseudomonas aeruginosa*	PQ762214
4	Strain K	*Bacillus spp*.	PQ761102
5	Strain L	*Bacillus cereus*	PQ761192

#### STRAIN H: *B. thuringiensis*

ACGTGTGTAGCCCAGGGCATAAGGGGCATGATGATTTGACGTCATCCCCACCTTCCTCCGGTTTGTCACCGGCAGTCACCTTAAAATGCCCAACTGAATGATGGCAACTAAAAACAAGGGTTGCGCTCGTTGCGGGACTTAACCCAACATCTCACGACACGAGCTGACGACAACCATGCACCACCTGTCACTCTGCTCCCGAAGGAAAAACCCTATCTCTAGGGTTGTCAGAAGATGTCAAGACCTGGTAAGGGTCTTCCCGTTGCTTCCAATTAAACCACATGCTCCACCGCTTGGGCGGGCCCCCGTCAATTCCTTTGAGTTTCAGCCTTGCGGCCGTACTCCCCAGGCGGAATGCTTAATGCGTTAACTTCAGCACTAAAGGGCGGAAACCCTCTAA.

#### STRAIN I *B.paramycoides*

ACAGTTTCTGTCACTTAGGCGGCTGGCTCCAAAGGGTACCCCACCGACTTCGGGTGTTACAAACTCTCGTGGTGTGACGGGCGGTGTGTACAAGGGCCGGGAACGTATTCACCGCGGCATGCTGATCCGCGATTACTAACGATTCCAGCTTCATGTAGGCGAATTGCAGCCTACAATCCGAACTGAAAACGGTTTTATGAAATTAGCTCCACCTCGCGGTCTTGCAGCTCTTTGTACCGTCCATTGTAACACGTGTGTAGCCCAGGGCATAAGGGGCATGATGATTTGACGTCATCCCCACCTTCCTCCGGTTTGTCACCGGCAGTCACCTTAAAATGCCCAACTGAATGATGGCAACTAAAAACAAGGGTTGCGCTCGTTGCGGGACTTAACCCAACATCTCACGACACGAGCTGACGACAACCATGCACCACCTGTCACTCTGCTCCCGAAGGAAAAACCCTATCTCTAGGGTTGTCAGAAGATGTCAAGACCTGGTA.

#### STRAIN J *P. aeruginosa*

AAGATCTCAAGGATCCCAACGGCTAGTCGACATCGTTTACGGCGTGGACTACCAGGGTATCTAATCCTGTTTGCTCCCCACGCTTTCGCACCTCAGTGTCAGTATCAGTCCAGGTGGTCGCCTTCGCCACTGGTGTTCCTTCCTATATCTACGCATTTCACCGCTACACAGGAAATTCCACCACCCTCTACCGTACTCTAGCTCAGTAGTTTTGGATGCAGTTCCCAGGTTGAGCCCGGGGATTTCACATCCAACTTGCTGAACCACCTACGCGCGCTTTACGCCCAGTAATTCCGATTAACGCTTGCACCCTTCGTATTACCGCGGCTGCTGGCACGAAGTTAGCCGGTGCTTATTCTGTTGGTAACGTCAAAACAGCAAGGTATTAACTTACTGCCCTTCCTCCCAACTTAAAGTGCTTTACAATCCGAAGACCTTCTTCACACACGCGGCATGGCTGGATCAGGCTTTCGCCCATTGTCCAATATTCCCCACTGCTGCCTCCCGTAAGAATCTGGACCGGGTCTCAGTTCCAGTGTGACTGAACATCCCTCAAACAATTACGGATCGTCGCCTTGGTAGGCCTTTACCCCCCCACTACCTAACCCAACCTAGGCCTATGAAAACGGGAGGTCCAAAAAACCCCCCTTTTTCCCCT.

#### STRAIN K *Bacillus* sp

CCTGTCAGTATCTGGTCCACCTTCGGCGGCTGGCTCCATAAAGGTTACCTCACCGACTTCGGGTGTTACAAACTCTCGTGGTGTGACGGGCGGTGTGTACAAGGCCCGGGAACGTATTCACCGCGGCATGCTGATCCGCGATTACTAGCGATTCCAGCTTCACGCAGTCGAGTTGCAGACTGCGATCCGAACTGAGAACAGATTTGTGGGATTGGCTTAACCTCGCGGTTTCGCTGCCCTTTGTTCTGTCCATTGTAGCACGTGTGTAGCCCAGGTCATAAGGGGCATGATGATTTGACGTCATCCCCACCTTCCTCCGGTTTGTCACCGGCAGTCACCTTAGAGTGCCCAACTGAATGCTGGCAACTAAGATCAAGGGTTGCGCTCGTTGCGGGACTTAACCCAACATCTCACGACACGAGCTGACGACAACCATGCACCACCTGTCACTCTGCCCCCGAAGGGGACGTCCTATCTCTAGGATTGTCAGAGGATGTCAAGACCTGGTAAGGTTCTTCGCGTTGCTTCGAATTAAACCACATGCTCCACC.

#### STRAIN L *B.cereus*

ACAGTTTCTGTCACTTAGGCGGCTGGCTCCAAAGGGTACCCCACCGACTTCGGGTGTTACAAACTCTCGTGGTGTGACGGGCGGTGTGTACAAGGGCCGGGAACGTATTCACCGCGGCATGCTGATCCGCGATTACTAACGATTCCAGCTTCATGTAGGCGAATTGCAGCCTACAATCCGAACTGAAAACGGTTTTATGAAATTAGCTCCACCTCGCGGTCTTGCAGCTCTTTGTACCGTCCATTGTAACACGTGTGTAGCCCAGGGCATAAGGGGCATGATGATTTGACGTCATCCCCACCTTCCTCCGGTTTGTCACCGGCAGTCACCTTAAAATGCCCAACTGAATGATGGCAACTAAAAACAAGGGTTGCGCTCGTTGCGGGACTTAACCCAACATCTCACGACACGAGCTGACGACAACCATGCACCACCTGTCACTCTGCTCCCGAAGGAAAAACCCTATCTCTAGGGTTGTCAGAAGATGTCAAGACCTGGTAAGGGTCTTCCCGTTGCTTCCAATTAAACCACATGCTCCACCGCTTGGGCGGGCCCCCGTCAATTCCTTTGAGTTTCAGCCTTGCGGCCGTACTCCCCAGGCGGAATGCTTAATGCGTTAACTTCAGCACTAAAGGGCGGAAACCCTCTAA.

## Discussion

**Fig. 15:**
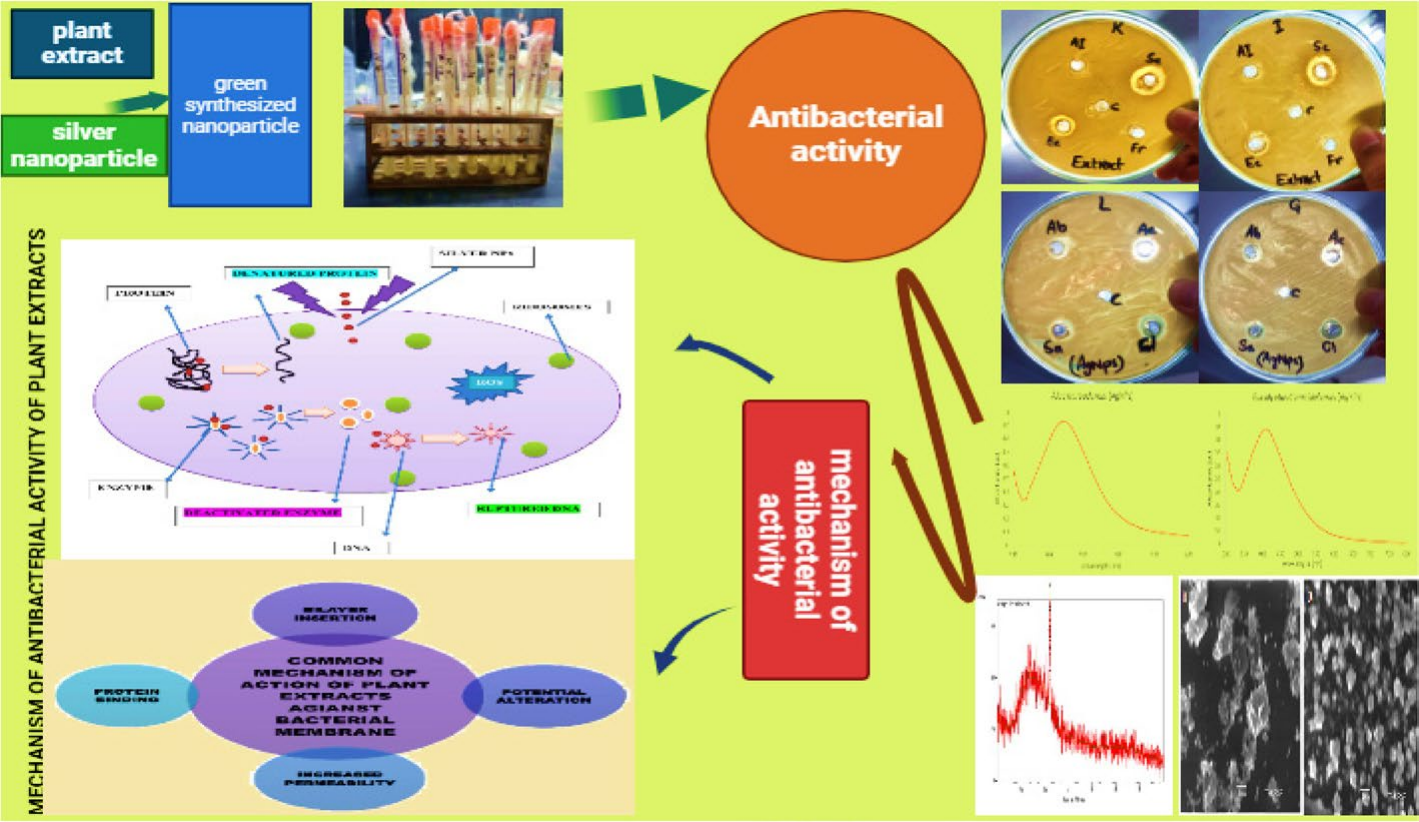
Schematic presentation of antibacterial activity and mechanism of antibacterial action of plant extracts and AgNPs on a bacterial cell

**Fig. 16:**
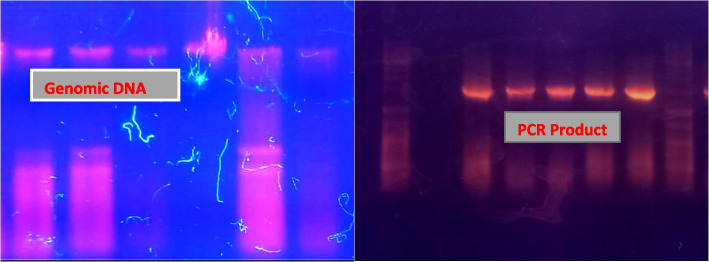
Genomic DNA isolation and PCR product of 16s rRNA gene (size 1500bp)

One major supply of antimicrobials is natural items. Identification of substances that function as suitable antibacterial agents has been the subject of extensive research [46]. Plant products containing phyto-chemicals and antibacterial agents are the most valuable resources for producing antibiotics that are both less harmful and more effective [37]. The previously mentioned plant extracts demonstrated noteworthy or moderate activities (9 mm to 14 mm) against clinical isolates of *B. thuringiensis, B. paramycoides, P. aeruginosa, B. coahuilensis* and *B. cereus.* In contrast, the plant extracts used in current study have shown excellent antibacterial activity results against the isolated pathogens from conjunctivitis. Conjunctivitis, which is typically brought on by resistant bacteria, can be treated and/or cured with these plant species used in current study Figs. 15 and 16.

Nsofor et al. [40] evaluated the aqueous and methanolic gel extracts of Aloe vera against gram-negative bacteria (*P. aeruginosa*) and gram-positive bacteria (*B. cereus*) by well diffusion method. According to Nsofor et al. [40], aqueous extract of Aloe vera showed the least antibacterial activity against *B. cereus* and *P. aeruginosa*. While ethanolic extracts of Aloe Vera displayed a high antibacterial activity against *B. cereus* and *P. aeruginosa*. However, in our present study, aqueous gel extract of Aloe vera showed no antibacterial activity against *P. aeruginosa* and *B. cereus*. Similar to this, there are several reports on the antibacterial effects of various Aloe vera kinds [2, 23].

The green synthesis of silver nanoparticles (AgNPs) is ideal because of its environmentally friendly approach. Employing different components of plants, bacteria, fungi, and algae offers an efficient, simple, and sustainable method for producing AgNPs. Plant extracts encompass a variety of biomolecules, including amino acids, proteins, enzymes, terpenes, alkaloids, flavonoids, phenols, tannins, and vitamins, all of which serve as reducing, capping, and stabilizing agents. In a similar manner, microorganisms produce a range of extracellular and intracellular biomolecules, including enzymes, amino acids, proteins, and numerous primary and secondary metabolites, which serve as reducing agents, capping agents, and stabilizing agents throughout the synthesis process. Green synthesized silver nanoparticles (AgNPs) exhibit maximum antibacterial properties against a wide range of both Gram-positive and Gram-negative bacteria. The current research study provides an examination of the green synthesis of AgNPs utilizing different plants against pathogenic bacterial isolates have been emphasized. Green synthesized nanoparticles with *C. limon* showed maximum ZOI of 16.16 mm against *B. thuringiensis* and minimum ZOI of 7.33 mm with *F. religiosa* against *B. cereus*. Almost all bacterial isolates showed resistance with no ZOI against green synthesized nanoparticles with *S. cumini* and *E. camaldulensis*. Green synthesized AgNPs of *S. aromaticum* aqueous extract showed antibacterial activity against all bacterial strains. They were most effective against *P. aeruginosa* and *B. paramycoides* while least effective against *B. thuringiensis.* Overall it can be observed that rapid, direct, and environmentally friendly synthesis methods utilizing plants and microbes demonstrate significant potential for AgNPs, although the precise mechanisms of synthesis and their antimicrobial modes of action remain unclear.

Aqueous and methanolic leaf extracts of *Syzygium cumini* (Jamun) exhibited antibacterial activity against gram-positive bacteria (*Bacillus subtilis* and *S. aureus*) and gram-negative bacteria, such as *P. aeruginosa* and *Salmonella* spp. Methanol extracts outperformed aqueous extracts in terms of potency [26]. In present study, aqueous leaf extract of *S. cumini* inhibited both gram-negative and gram-positive bacteria. Antibacterial activity was maximum against *B. coahuilensis* and minimum against *P. aeruginosa* and *B. paramycoides*. According to Gowri & Vasantha [26], flavonoids, alkaloids, steroids, glycosides, tannins, phenols, and saponins were abundant in the leaves of *S. cumini*.

Tshabalala et al. [39] and Patel et al. [45] found that clove exhibit high antibacterial activity. Furthermore, Elisha et al. [22] prepared cold water, hot water and ethanolic extracts of *S. aromaticum* to observe the antibacterial activity against different bacterial isolates including *S. typhi* by disc and well diffusion method. The plant extracts showed antibacterial activity against all tested isolates, including *S. typhi*. In present research study, aqueous extract of *S. aromaticum* (clove) showed maximum antibacterial results with ZOI of 10 mm against *B. thuringiensis* only while other bacterial strains were resistant to it. All bacterial isolates were resistant against plant extracts of *A. indica*, *F. religiosa* and *A. barbadensis*.

In other studies, ethanolic and aqueous leaf extracts of *F. religiosa* were shown to have antibacterial action against pathogenic bacterial strains such as *B. subtilis*, *E. coli*, *S. aureus*, and *P. aeruginosa* and *S. typhi*. The leaf extracts varyingly inhibited most of the examined bacteria [52]. While in this present research, aqueous leaf extract of *F. religiosa* did not show any antibacterial effect against any of bacterial strains. On contrary, green synthesized AgNPs of *F. religiosa* aqueous leaf extract showed antibacterial activity against all bacterial strains. Maximum inhibitory activity was against *P. aeruginosa* with ZOI of 13 mm and minimum antibacterial activity was with ZOI of 7.33 mm against *B. cereus*.

Abd & Hasan [1] proved that silver nanoparticles of Aloe Vera leaf extract were effective against both gram-positive (*S. epidermidis*) and gram-negative (*P. aeruginosa*) bacteria. AgNPs solutions were prepared in different concentrations (12.5, 25, 50 and100) mg/ml were evaluated against antibiotic resistant bacterial isolates. With maximum inhibition zone against *S. aureus*, *S. epidermidis*, *A. baumannii* and *P. aeruginosa* were (22, 23, 20 and 21) mm respectively at concentration (100) mg/ml and the minimum zone at concentration (12.5) mg/ml at the same isolates were (12, 11, 10 and10) mm respectively. While, in comparison, green synthesized AgNPs of *A. babadensis* (Aloe Vera) showed antibacterial activity against all gram-positive bacteria but there was no activity against gram-negative bacteria (*P. aeruginosa*) in present work. *P. aeruginosa* was resistant against green synthesized silver nanoparticles with *A. barbadensis*. In related study, Anavil et al. [9] exhibited that silver nanoparticles of *A. barbadensis* showed maximum antibacterial activity against *E. coli* than *S. aureus. *Rossos et al. [49] demonstrated that silver nanoparticles of eucalyptus leaves extract were highly active against *P. aeruginosa*, *S. epidermidis* but they performed poorly against *S. aureus*. In this present work, these AgNPs were slightly active against *B. cereus* while no activity was observed against other bacterial strains including *P. aeruginosa, B. thuringiensis, B. paramycoides* and *B. coahuilensis*.

Similarly, antibacterial properties of lemon (*C. limon*) have been determined against variety of bacteria. *C. limon* alcoholic extracts showed ZOIs of 15 mm and 20 mm against *Escherichia coli* and *Streptococcus pyogenes* respectively. The ethanolic and methanolic extracts of *C. limon* peel showed ZOIs of 5 mm and 6 mm against *E. coli* respectively. On the other hand, there was no inhibition effect of both alcoholic extracts on *Streptococcus pyogenes* [5, 50]. In present study, results showed that aqueous extract and green synthesized AgNPs of *C. limon* showed antibacterial activity against all identified bacterial strains with maximum ZOI of 16.16 mm and minimum ZOI of 10.83 mm. Other than *C. limon*, *A. indica. F. religiosa* and *S. aromaticum* showed antibacterial activity against all identified bacterial strains. Aqueous extracts of some plants such as *A. indica*, *F. religiosa* and *A. barbadensis* and AgNPs of plant *S. cumini* didn’t exhibit any antibacterial activity against isolated identified bacterial strains. The medicinal plant species included in this study that showed efficacy against the bacterial isolates may be studied in more detail to find naturally occurring bioactive compounds.

## Conclusion

Significant antibacterial activity against isolated bacterial strains has been demonstrated by a few aqueous plant extracts (*C. limon, S. cumini, E. camaldulensis, A. cepa,* and *S. aromaticum*) and green synthesized silver nanoparticles (*A. indica, F. religiosa, C. limon, S. aromaticum, A. cepa,* and *A. barbadensis*). Due to the adverse effects that chemically manufactured antibiotics have on the human body at high dosages. Antibiotic medication may be replaced by biological antibacterial tools such as plant extracts and green synthesized silver nanoparticle formulations. In order to prepare these antibacterial agents for their therapeutic use against ocular infections, additional study on them can be undertaken. Various aspects should be taken into account for the future production of AgNPs using plants or microorganisms. Initially, it is important to choose the appropriate plants or microbes that allow for a straightforward, quick, and environmentally friendly synthesis process. When selecting plants, researchers need to take into account the accessibility of the plants and the simplicity of their extraction methods. The chosen plants should be readily obtainable, and the extraction process must be straightforward to facilitate the large-scale production of AgNPs. In the same manner, scientists ought to prioritize non-pathogenic and fast-growing microbes to ensure safety and ease of handling throughout the synthesis process. In this context, probiotic microbes could serve as excellent synthetic agents. Secondly, the analysis of biomolecules found in plant extracts, microbial biomass, or culture supernatants is conducted. It is thought that various biomolecules found in plant extracts or microbial culture supernatants play a key role in the synthesis and stabilization of silver nanoparticles (AgNPs). The functions of different enzymes in biosynthesis require thorough investigation. Furthermore, these biomolecules contribute to improving the antibacterial effectiveness of synthesized AgNPs. Consequently, it is essential to explore the biomolecules found in plant extracts or microbial culture supernatants to ensure the successful synthesis of AgNPs. Third, optimizing parameters is crucial for the rapid, stable, and large-scale production of AgNPs. Numerous studies have indicated that factors like the concentration of plant extract and AgNO3, incubation time and temperature, and the pH of the reaction significantly influence the synthesis process. As a result, optimizing these reaction conditions can facilitate mass production on an industrial scale. Additionally, research into the antibacterial mechanisms is essential [8].

### Future perspectives

The majority of studies has highlighted the effectiveness of AgNPs at a preliminary screening level but has not explored the specific mechanisms involved. Determining the mechanism by which AgNPs act against pathogens is crucial. Additionally, it is essential to explore the cytotoxic effects of biosynthesized AgNPs on human cells. Several studies have indicated that AgNPs can have cytotoxic effects on human cells. Therefore, it is crucial to examine the possible toxicity of green synthesized AgNPs on healthy human cells to assurance their safe application for both human health and the environment.

## Data Availability

No datasets were generated or analysed during the current study.
